# Validity of Social Cognition Measures in the Clinical Services for Autism Spectrum Disorder

**DOI:** 10.3389/fpsyg.2020.00004

**Published:** 2020-02-05

**Authors:** Maria Chiara Pino, Francesco Masedu, Roberto Vagnetti, Margherita Attanasio, Chiara Di Giovanni, Marco Valenti, Monica Mazza

**Affiliations:** ^1^Department of Applied Clinical Sciences and Biotechnology, University of L’Aquila, L’Aquila, Italy; ^2^Regional Centre for Autism, Abruzzo Region Health System, L’Aquila, Italy

**Keywords:** autism spectrum disorder, diagnostic process, receiver operating characteristic (ROC) curve, social cognition, Theory of Mind, clinical utility

## Abstract

The current study evaluated three social cognition (SC) tests for their clinical utility in aiding autism diagnosis. To do so, we compared the performance of 86 children with autism spectrum disorder (ASD) and 68 typically developing (TD) children, all aged from 4 to 10 years old, on three SC tasks [the Social Information Processing Interview (SIPI), the Comic Strip Task (CST), and the children’s version of the Eyes Task] and calculated threshold scores that best differentiated the two groups. While difficulties in these abilities appear to represent the “central core” of ASD, services have largely ignored SC tests when supporting autism diagnoses. Therefore, this study attempted to validate and evaluate the diagnostic potential of these three tasks for children with ASD. To investigate the accuracy of these SC tests, we used the receiver operating characteristic (ROC) curve. As expected, the ASD group performed worse than the TD group on the SIPI and CST, but contrary to our prediction, the groups did not significantly differ on the Eyes Task. Specifically, the overall area under the curve (AUC) for the SIPI was 0.87, with a sensitivity of 73.5% and a specificity of 83.9% at the best cutoff point (score range 0–36; best cutoff = 31). The overall AUC for the CST was 0.75, with a sensitivity of 71.1% and a specificity of 77.0% at the best cutoff point (score range 0–15; best cutoff = 11). The overall AUC for the Eyes Task was 0.51, with a sensitivity of 50.3% and a specificity of 40.2% at the best cutoff point (score range 0–54; best cutoff = 45). In conclusion, the results showed that the SIPI test has good predictive power for classifying children with ASD. It should provide substantial supplementary clinical information and help to consolidate diagnostic procedures based on standard tools. Moreover, the results of the study have substantial implications for clinical practice: the better the knowledge of SC functioning in children with ASD, the more effective the intervention program for rehabilitation.

## Introduction

Autism spectrum disorder (ASD) is a neurodevelopment condition characterized by deficits in two domains: (1) social communication and social interaction and (2) restricted, repetitive patterns of behaviors, interests, or activities (American Psychiatric Association, 2013). A large body of research supports the hypothesis that difficulties in social interaction and communication can be explained by a deficit in social cognition (SC) abilities (Happé and Frith, 2014; Lai et al., 2014; Mazza et al., 2017; Pino et al., 2017). SC is a set of cognitive abilities involved in the processing and interpretation of the social world (Mazza et al., 2010; Bishop-Fitzpatrick et al., 2017; Pino et al., 2017). A main component of SC is the Theory of Mind (ToM), namely the ability to understand the mental and emotional states of other people (Mazza et al., 2014); it affects the development of social behavior from birth. A crucial development of ToM occurs around 3–4 years of age, when children acquire false belief attributions and realize that mental states, such as beliefs or the intentions of other people, may not be true (Mazza et al., 2017). Thus, ToM deficits are related to social communication and social interaction criteria of the *Diagnostic and Statistical Manual of Mental Disorders, Fifth Edition* (DSM-5).

Several studies (Shamay-Tsoory and Aharon-Peretz, 2007; Shamay-Tsoory et al., 2010; Mazza et al., 2014; Baron-Cohen et al., 2015; Pino et al., 2017) suggest that ToM is not a unitary construct; rather, it involves two distinct components: cognitive and affective. Specifically, the cognitive component of ToM includes the ability to understand what other people are thinking and make inferences about their beliefs, intentions, and motivations. The affective ToM component is the ability to understand what other people are feeling in a specific emotional context and comprehend their emotions. Understanding another person’s cognitive or affective state is a crucial ability for development and production of adequate social behaviors (Krebs and Russell, 1981; Hoffman, 1984; Batson, 1987; Mazza et al., 2017).

According Happé and Frith (2014), social behavior develops around 5 years of age, when children are able to differentiate their own internal states form those of others (Mazza et al., 2017). Children with ASD show difficulties in understanding other people’s mental state and their perspectives, and this deficit might compromise social behavior development (Frith and Happé, 1994; Happé, 1994; Frith and Frith, 2003; Jones et al., 2010; Mazza et al., 2014; Ziv et al., 2014).

The ToM hypothesis of ASD was first introduced by Baron-Cohen et al. (1985) three decades ago; it demonstrates difficulties for children with ASD in passing false belief tasks. Recent studies suggest that adults with ASD have difficulties in implicit mentalization tasks (measured by spontaneous looking patterns), despite the fact that they can pass classic explicit mentalizing tasks (direct questions about others mental states; Jones et al., 2018). Differentiation between the theoretical ToM components is crucial for future research in ASD (Altschuler et al., 2018).

Some mentalizing tests, such as the Eyes Test (Baron-Cohen et al., 1997, 2001), require emotion recognition to infer mental states (Jones et al., 2018). This test should reflect the mentalizing process and the ability to understand other’s mental states, such as emotions, thoughts, desires, beliefs, and goals (Peterson and Slaughter, 2009; Franco et al., 2014). Children and adults with ASD present lower performance on the Eyes Test (Baron-Cohen et al., 2001; Franco et al., 2014). Specifically, individuals with ASD have difficulties in processing information from the faces of others, such as facial expression and eye gaze, which play a significant role in SC (Hadjikhani et al., 2004; Pellicano et al., 2007; Ramachandran et al., 2010; Franco et al., 2014).

Deficits of social interaction in individuals with ASD are not related to general intellectual functioning. Rather, they are specific to the SC competences (Baron-Cohen et al., 1997; Ziv et al., 2014; Mazza et al., 2017). Ziv and Sorongon (2011), following Crick and Dodge’s (1994) social information processing model, suggested that many mental steps occur before individuals implement a behavioral response to social cues, such as the encoding of social cues, interpretation of the cues, clarification of goals, generation of a behavioral response, response construction, response decision, and realization of the behavior response (Crick and Dodge, 1994; Ziv and Sorongon, 2011; Ziv et al., 2014; Mazza et al., 2017). According to this model, these internal processes include the ability to understand thoughts, intentions, and feelings of others (ToM) and select the adequate social responses (Crick and Dodge, 1994). Subsequently, Ziv et al. (2014) showed deficits in social information processing abilities in preschool children with ASD using the Social Information Processing Interview (SIPI), an instrument that allows one to evaluate social behavior and the pattern of social information processing based on Crick and Dodge’s (1994) model. Ziv et al. (2014) demonstrated that children with ASD had a specific difficulty in social information processing; the ToM deficits were related to inadequate social behavior and poor social communication skills (Lerner et al., 2011; Ziv et al., 2014; Mazza et al., 2017). According to Mazza et al. (2017), social behavior is a consequence of how children process social cues. Considering that severe difficulties in social interaction are a defining feature of individuals with autism (Fletcher-Watson et al., 2014; Mazza et al., 2017), the SC assessment in ASD individuals, including psychometric evaluation of commonly used SC tasks, might help clinicians collect additional information and plan the best treatment in ASD research (National Advisory Mental Health, 2016; Morrison et al., 2019).

In ASD research, the SC construct is widely investigated, but it is rarely considered in the clinical practice due to a lack of well-validated tests with established psychometric data, as highlighted by Morrison et al. (2019). In contrast, the use of an SC test in ASD services might improve the diagnostic process and be exceedingly useful for prognoses and creating specific rehabilitation treatments for different age groups. Thus, the aim of the present study was to evaluate three SC tests for their clinical utility in aiding autism diagnosis. We compared performance by ASD and typically developing (TD) children on three SC tasks. Specifically, we chose to use the SIPI (Ziv and Sorongon, 2011; Ziv et al., 2014) for evaluation of social information process abilities, the Comic Strip Task (CST, Cornish et al., 2010; Sivaratnam et al., 2012) to assess the ToM sub-components (beliefs, emotions and intentions), and the children’s version of the Eyes Task to evaluate the ability to understand and infer mental and emotional states regardless of the child’s language level. For each test, we calculated threshold scores that best differentiated the two groups using the receiver operating characteristic (ROC) curve.

## Materials and Methods

### Participants

One hundred-fifty-four children participated in this study: 86 children with ASD (75 males and 11 females, from 4 to 10 years old, recruited by the Reference Regional Centre for Autism in L’Aquila in the Abruzzo Region, Italy) and 68 TD children (60 males and 8 females, from 4 to 10 years old). The TD children were recruited from a nursery (for 4- to 5-year-old children) and a primary school (for 6- to 10-year-old children) located in L’Aquila. We chose to match the two groups by verbal mental age (VMA), as assessed by the Test for Reception of Grammar (TROG-2; Bishop, 2003). Differences between the two groups emerged for chronological age, where ASD children (mean = 7.64 years, SD = 1.53) were older than TD children [mean = 6.62 years, SD = 1.79; *t*(152) = 3.81, *p* < 0.001] but did not differ in VMA [ASD: mean = 6.96 years, SD = 2.35; TD: mean = 7.52 years, SD = 2.47; *t*(152) = 1.43, *p* = 0.15]. The exclusion criterion was intellectual disability; the participants had an IQ > 80.

The ASD sample comprised children who came for a first-time diagnosis as well as those who came for a second evaluation. All previously diagnosed ASD children received special education through a support teacher. They also followed therapies provided by the National Health System: speech therapy, psychomotor intervention, and Applied Behavioral Analysis.

The clinical process for ASD diagnosis commences with an experienced neuropsychiatrist who observes the child and interviews caregivers. Thereafter, an experienced psychologist performs the Autism Diagnostic Observation Schedule–Second Edition (ADOS-2; Lord et al., 2012). Finally, they consult with one another to make the ASD diagnosis according to the DSM-5 (American Psychiatric Association, 2013) criteria and ADOS-2 outcomes. Clinicians directly involved in the clinical practice participated in the study. ASD participants were level 1, according to DSM-5 criteria (American Psychiatric Association, 2013): most of them showed a delayed language development. ADOS-2 comparison scores of our sample ranged from low to moderate autism-related symptoms. None of the participants had comorbidities with other disorders. All the children were native Italian speakers.

### Procedure

This study was performed in accordance with the recommendations of the Ethics Committee of Local Health Unit 1 (ASL1-Avezzano, Sulmona, L’Aquila), Abruzzo Region, L’Aquila, Italy. The Ethics Committee approved the protocol (number 186061/17) prior to the recruitment of participants, according to the principles established by the Declaration of Helsinki. Informed consent from the child and her or his parents was obtained before participation. Children with ASD were tested at the Reference Regional Centre for Autism, Abruzzo Region Health System, L’Aquila, Italy, whereas TD children were tested in their nurseries or schools. All children were tested individually by an expert psychologist in a quiet room according to the principles established by the Declaration of Helsinki.

### VMA Measure

According to recent literature (Pino et al., 2017), children with ASD show a delay in developing SC abilities based on chronological age, whereas VMA seems to be a good predictor of ToM abilities (Happé, 1994; Pino et al., 2017). Moreover, social difficulty does not appear to be based on the general IQ level, whereas VMA appears to be a more promising associated measure (Pino et al., 2017, 2018).

The literature suggests that children with ASD can use verbal strategies to support their reasoning during ToM tasks (Happé, 1995; Durrleman et al., 2019). Grammatical skills are particularly important during mentalizing (Fisher et al., 2005; de Villiers, 2007; Milligan et al., 2007). For these reasons, we chose to match two groups based on VMA, as assessed with the TROG-2 (Bishop, 2003), a standardized measure of receptive language that allows one to evaluate the ability to understand verbal language. The TROG-2 evaluates the comprehension of grammatical structures and contrasts grammatical indicated by suffixed, functional words, and order word. The test examines 20 syntactic constructions, each of which is examined with a block of four items. Participants select the picture–out of four presented choices–that corresponds to the sentence read by examiner. Standard and age-equivalent scores are made by the total number of blocks passed.

### SC Measures

#### SIPI

The SIPI (Ziv and Sorongon, 2011; Ziv et al., 2014) is a 20-min structured interview based on a storybook-easel that depicts a series of vignettes in which a protagonist is either rejected by two other peers or provoked by another peer. Each type of vignette is combined with each type of peer intent to generate four stories: (1) a non-hostile peer-entry rejection story, (2) an ambiguous peer-entry rejection story, (3) an accidental provocation story, and (4) an ambiguous provocation story. According to Ziv et al. (2014), the scores correspond to four of the five mental steps of social information-processing proposed by Crick and Dodge’s (1994) model: (1) encoding, (2) interpretation of cues, (3) response construction, and (4) response evaluation.

An example of a SIPI story is the following: Michael is watching the other children playing. Michael walks up to the other children and asks them: “Can I play with you?” The child says: “Sorry. The teacher said only two can play in the block area” (for details, see Ziv et al., 2014).

The Encoding component evaluates the level of detail that the child recalls across the four stories. Thus, the examiner asks the child: “Tell me what happened in the story, from the beginning to the end.” A code of 0 is given to children who recall no correct details from the stories and a code of 1 to children who correctly recall all the details in all the stories. An overall score is then calculated (ranging from 0 to 4).

The Interpretation component evaluates hostile attribution to others’ behavior (the question is: “Do you think the other children who didn’t let Michael play are mean or not mean?”). The answers are coded with 0 or 1, and an overall score (0–4) is then calculated, with higher scores representing higher levels of hostile attribution bias. Scores for this component are inversely encoded compared with the other SIPI components; that is, a higher score indicates a major tendency to consider the behavior of other children as hostile.

The Response Generation score is derived from the child’s responses to the open-ended question: “Pretend that you ask your friends if you can play with them and they say that only two can play in the block area. What would you do?” For each story, the examiner encodes the response as competent or non-competent and assigns a code of 1 if the child’s response is classified as competent and of 0 if the answer is classified as non-competent. An overall score (from 0 to 8) is then calculated.

The Response Evaluation items examine the way in which the child assesses the behavior of other people as being right or wrong. This score is obtained by combining the 36 response evaluation questions (4 stories × 3 presented responses × 3 questions per presented response). The three response variables for these steps are: (1) a competent response (e.g., Michael could say, “Then can I play next?”); (2) an aggressive response (e.g., Michael could kick apart the blocks and say to the other children, “If I can’t play, then you can’t play either!”); and (3) an avoidant or inappropriate response (e.g., Michael could cry and say, “It’s not fair”; Pino et al., 2018). The total number of non-competent responses (aggressive and avoidant responses) are subtracted from the total number of competent responses and adjusted for negative scores in order to obtain a score (from 0 to 36).

For the purpose of this study, we also calculated a total score. In our analysis, we did not include the Encoding subscale because one item showed poor psychometric properties (Ziv and Sorongon, 2011). Instead, we used the three main SIPI scores as reported by Ziv and Sorongon (2011): Interpretation, Response Generation, and Response Evaluation. A higher score on the Interpretation subscale (range 0–4) represents hostile attribution. Therefore, we first converted this scale into a non-hostile attribution scale (called Positive Interpretation) by calculating its complementary scale using the following formula: 4 − the number of hostile responses. Next, we summed the Positive Interpretation, Response Generation, and Response Evaluation scores to obtain a total SIPI score.

We decided to use the SIPI because it can evaluate the social cue processing that is closely related to the ability to understand and recognize the intentions, beliefs, and emotions of other people (ToM). According to Mazza et al. (2017), if a child has difficulties in processing social cues within a context, she or he will show difficulties in the ability to evaluate whether other people’s social behavior is right or wrong and she or he will respond inadequately in social situations. This phenomenon will impair social relations with others. The test is coded by considering different aspects of the social information process, including the hostile style of attribution and the generation of socially competent, avoidant, or hostile responses. This factor represents an added value in the diagnostic evaluation; in fact, during the assessment, some behavioral problems may arise that should be considered for future intervention or evaluation. Indeed, Ziv and Sorongon (2011) demonstrated that preschoolers with aggressive tendencies evaluate aggressive responses as better ones. However, future research should deepen this aspect in the clinical setting for details see Supplementary Material.

#### CST

The CST (Cornish et al., 2010; Sivaratnam et al., 2012) is a 21-item measure that was developed to assess three aspects of ToM: understanding Beliefs, Intentions, and Emotions. There are five items in each component, and each comprises a five-picture comic strip that illustrates everyday social scenarios involving interpersonal interactions that are familiar to young children. Each component has a maximum score of 5, with a total test score range of 0–15 (higher scores correspond to better ToM). We used the CST because it does not require verbal abilities, a factor that allows one to measure ToM deficits *per se.* Moreover, the CST is suitable for a wide swath of the ASD population; it was designed for 4- to 8-year-old children, but it can be used in both younger and older children (Philpott et al., 2013). We also suppose that the use of comics might attract the attention of children, and the formal administration is very brief (10–15 min; Sivaratnam et al., 2012).

#### Eyes Task–Children’s Version

The Eyes Task (Franco et al., 2014) consists of a series of black and white photos of children’s eyes; they portray either mental states or primary emotions. The expressions selected as primary emotions were happy and surprised (positive/neutral valence) and sad and angry (negative valence), while excited and thinking (positive/neutral valence) and worried and shy (negative valence) were selected to represent mental states (for further details, see Franco et al., 2014; Pino et al., 2017). A total of 56 images are presented to the child; each represents one of the stimuli described above with two possible responses. If the child responds correctly, the item is coded as 1; otherwise, it is coded as 0. A total score is then calculated by adding the correct responses to the primary emotions and mental states. Total scores range from 0 to 56 (with higher scores indicating better ToM performance). We used the version by Franco et al. (2014) because stimuli are derived from naturalistic pictures of children rather than posed adults like the version of Baron-Cohen et al. (2001). Moreover, the Eyes Task (Franco et al., 2014) requires fewer cognitive demands because it shows one eye picture with two possible responses. This design is suitable even for low-functioning autism. Score calculations for each test are shown in Table 1.

**TABLE 1:** Score construction.

	Score Construct	Count (#)	Range
*SIPI*	**Interpretation**		
	*Score*_*I^–*_ (Negative interpretation)	#(Hostile responses)	0–4
	*Score*_*I^+*_ (Positive interpretation)*	4-#(Hostile responses)	0–4
	**Response generation**		
	*Score*_*G*_	#(Competent responses) + [4-#(Non-competent responses)]	0–8
	**Response evaluation**		
	*Score*_*E*_	#(Competent responses) + [24-#(Non-competent responses)]	0–36
	**Total SIPI Score**		
	*Score*_*SIPI*_	*Score*_*I^+*_ + *Score*_*G*_ + *Score*_*E*_	0–48
*Comic Strip Task*	**Intention**		
	*Score*_*i*_	#(Correct responses)	0–5
	**Beliefs**		
	*Score*_*b*_	#(Correct responses)	0–5
	**Emotions**		
	*Score*_*e*_	#(Correct responses)	0–5
	**Total CST Score**		
	*Score*_*CST*_	*Score*_*i*_ + *Score*_*b*_ + *Score*_*e*_	0–15
*Eyes Task*	**Primary emotions**		
	*Score*_*P*_	#(Correct responses)	0–28
	**Mental states**		
	*Score*_*M*_	#(Correct responses)	0–28
	**Total Eyes Task Score**		
	*Score*_*ET*_	*Score*_*P*_ + *Score*_*M*_	0–56

*This score is used to calculate the total SIPI score.

### Data Analysis

#### Descriptive Analysis

Demographic parameters and total scores for the SIPI, the CST, and the Eyes Task were recorded for both groups (ASD and TD).

#### Reliability and Internal Consistency

We assessed the internal consistency and reliability, in relation to the overall measure, for each ToM measure (the SIPI, the CST and the Eyes Task) using Cronbach’s α.

#### ROC Analysis

The overall goal of the ROC analysis was to estimate the cutoff points for the ToM measures that could distinguish between the two groups. ROC analysis is used to assess the diagnostic properties of tests, specifically, to assess the way in which various measures generally discriminate between categories of subjects. In order to do this, a cutoff point must be established. Based on the cutoff point, we can determine whether a person with a certain score belongs to one category or another (e.g., normal/non-clinical or clinical group). ROC analysis can also be used when comparing the diagnostic performance of two or more tests (Westin, 2001).

In a ROC curve, the true-positive rate (sensitivity) is plotted as a function of the false-positive rate (100 - specificity) for various cutoff points. The obtained area under the curve (AUC) signifies how well a parameter distinguishes between two groups. In order to establish a diagnostic threshold and corresponding test sensitivity and specificity, we established the cutoff as the value where the highest percentage of true positives was correctly classified as positive and true negatives was correctly classified as negative (Cleves, 1999). In our study, ROC curve analysis was performed to evaluate the accuracy of the total score of ToM measures (the SIPI, CST, and children’s version of the Eyes Task) in discriminating between ASD and TD children, using ADOS-2 and DSM-5 criteria as the *gold standard*. The analysis was performed using STATA version 14 statistical software (StataCorp, 2015).

#### Optimizing Diagnostic Performance

To improve diagnostic performance, we constructed a test based on a linear combination of the SIPI, CST, and Eyes Task scores. A multivariate logistic regression was performed to obtain the respective logit scores. The logit model allowed us to assess the marginal diagnostic advantage of the SIPI, CST, and Eyes Task and test their statistical significance. Their marginal diagnostic gain can be viewed in terms of the AUC of the ROC curve of the new logit score.

## Results

### Descriptive Analysis

Compared with TD children, children with ASD scored significantly lower on the SIPI [*t*(152) = 9.19, *p* < 0.001] and the CST [*t*(152) = 5.59, *p* < 0.001], but they recorded similar scores on the Eyes Task [*t*(152) = 0.43, *p* = 0.66]. The results are shown in Table 2.

**TABLE 2:** Between-group differences for demographic data, clinical information, and social cognition measures.

	ASD (*N* = 86) Mean (SD)	TD (*N* = 68) Mean (SD)	*t* (df = 152)	*p*
Chronological age (in years)	7.64 (1.53)	6.62 (1.79)	3.81	<0.001*
Verbal mental age (in years)	6.96 (2.35)	7.52 (2.47)	1.43	0.15
ADOS-Social communication and social interaction	8.34 (3.50)	–	–	–
ADOS-Repetitive and stereotyped behaviors	1.26 (1.12)	–	–	–
ADOS total scores	9.78 (3.62)	–	–	–
*Social cognition measures (total score)*				
SIPI	22.3 (9.22)	34.3 (6.13)	9.19	<0.001*
CST	9.01 (2.48)	11.2 (2.22)	5.59	<0.001*
Eyes Task	44.3 (8.01)	43.6 (12.0)	0.43	0.66

*Significant difference for *p* < 0.05.

### Internal Consistency Results

The results for the CST demonstrated high internal consistency (Cronbach’s α = 0.80), the results for SIPI demonstrated good internal consistency (Cronbach’s α = 0.76), and the results for Eyes Task demonstrated high internal consistency (Cronbach’s α = 0.80).

### ROC Analysis

**FIGURE 1:**
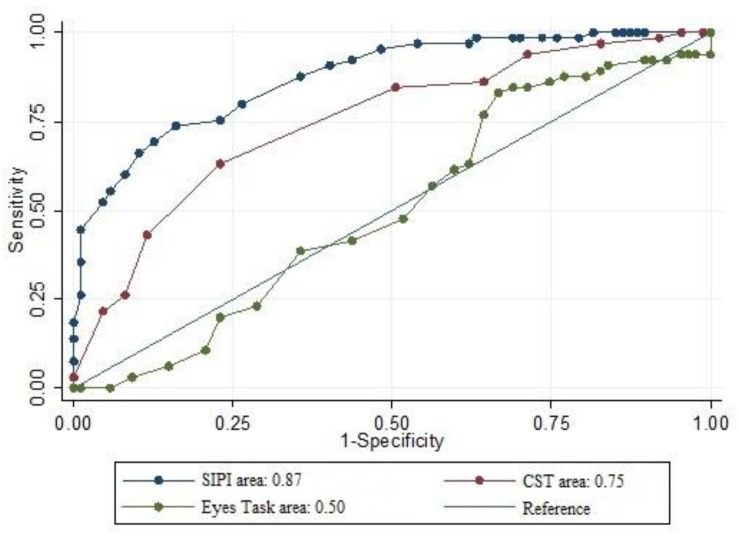
Comparison of ROC curves of SC measures (SIPI, CST, and Eyes Task) with relative AUCs.

For the SIPI, the overall AUC_*SIPI*_ was 0.87 (*SE* = 0.02). The optimal cutoff value was 31 (correctly classified = 79.3%), which corresponded to a sensitivity of 73.5% and a specificity of 83.9%. For the CST, the overall AUC_*CST*_ was 0.75 (*SE* = 0.03), and the optimal cutoff value was 11 (correctly classified = 71.1%). This value corresponded to a sensitivity of 63.0% and a specificity of 77.0%. For the ET, the overall AUC_*ET*_ was 0.51 (*SE* = 0.04). The optimal cutoff value was 45 (correctly classified = 50.3); this value corresponded to a sensitivity of 63.24% and a specificity of 40.2%. The analysis revealed a significant difference between AUC measures (χ^2^ = 60.9, *p* < 0.001). The results are reported in Table 3, and the ROC curves are displayed in Figure 1.

**TABLE 3:** ToM measures’ AUCs and cut-offs with respective sensitivity and specificity.

Social cognition measures	AUC*	SE	95% CI	Cutoff	Sensitivity(%)	Specificity (%)	Correctly Classified (%)
SIPI	0.87	0.02	0.81–0.92	31	73.5	83.9	79.4
CST	0.75	0.03	0.67–0.82	11	63.1	77.0	71.1
Eyes Task	0.51	0.04	0.42–0.60	45	63.2	40.2	50.3

*Comparison between AUC show a significant difference (χ^2^ = 60.9, *p* < 0.001).

### Diagnostic Performance Optimization

**FIGURE 2:**
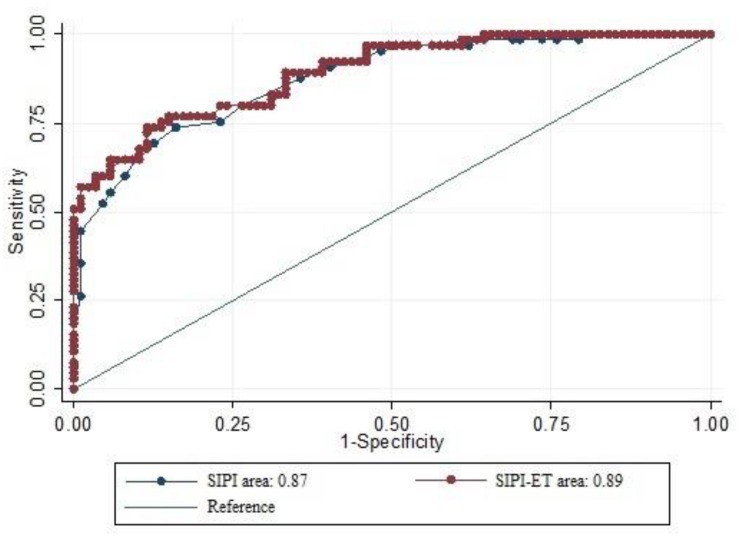
Comparison of ROC curves of SIPI and SIPI-ET with relative AUCs.

The logistic model showed that the SIPI (β = 0.26, *SE* = 0.04, *z* = 5.23, *p* < 0.001) and Eyes Task (β = -0.10, *SE* = 0.03, *z* = -3.12, *p* < 0.001) were statistically significant diagnostic predictors, while the CST (β = 0.18, *SE* = 0.11, *z* = 1.66, *p* = 0.09) was not. When merging the two tests into one new test (hereafter referred to as SIPI-ET), we observed an improvement in overall diagnostic performance (AUC_*SIPI–ET*_ = 0.89, *SE* = 0.02). However, there was no statistically significant difference between AUC_*SIPI–ET*_ and AUC_*SIPI*_ (χ^2^ = 2.39, *p* = 0.12), a finding that indicates that there was no statistically significant improvement. Figure 2 shows AUC_*SIPI*_ versus AUC_*SIPI–ET*_.

## Discussion

This study highlights the utility of including a SC battery of tests to improve the quality and quantity of information collected during procedures for diagnosing ASD. According to Lai et al. (2014), social difficulties in children with autism have been reported since 1985, when it was first highlighted by Baron-Cohen and collaborators. This impaired ability is believed to play a central role in the social communication and interaction deficits (the first diagnostic criterion in DSM-5; American Psychiatric Association, 2013) of ASD individuals. In fact, this criterion requests clinicians to evaluate abilities as “reduced sharing of interest, emotion or affect” (criterion A1/DSM-5; American Psychiatric Association, 2013); “deficits in social–emotional reciprocity” (criterion A1/DSM-5; American Psychiatric Association, 2013), “deficits in non-verbal communicative behaviors used for social interaction” (criterion A2/DSM-5; American Psychiatric Association, 2013), and “deficits in developing, maintaining, and understanding relationships” (criterion A3/DSM-5; American Psychiatric Association, 2013). All of these competences are part of the complex cognitive construct of SC. Despite the significant role exerted by SC components, such as ToM, in ASD diagnoses, assessment of these competences is neglected in Italian clinical services. Indeed, the use of ToM tests is limited to the research field. For this reason, we evaluated the accuracy of SC measures–using an ROC curve–to discriminate ASD from TD children in a small Italian sample. Additionally, we determined the best cutoff point for the three SC measures used: the SIPI, CST, and Eyes Task.

The results of the ROC analysis showed that the SIPI had good predictive power in terms of accurately classifying children with ASD. On the other hand, the CST showed moderate predictive power, while the Eyes Task showed no ability to correctly distinguish between ASD and TD.

Regarding the Eyes Task, Franco et al. (2014) found that ASD were less accurate compared to TD children, but based on our results, the difference between the groups would not allow us to characterize the ASD individuals during the diagnostic process. In fact, ASD children around 5–6 years old can recognize simple emotional and mental states (i.e., happy, sad, angry, and worried). Thus, there were no distinguishing characteristics in their performance compared to their TD peers. For the CST, the original authors administered the test to 4- to 8-year-old children with high functioning ASD (Sivaratnam et al., 2012). They performed significantly worse compared to controls on the overall two-subscale CST (belief- and intention-understanding). There were no group differences in the emotion understanding subscale performance (Cornish et al., 2010; Sivaratnam et al., 2012). In our study, unlike the authors of CST, we matched subjects by VMA. This method reduced differences in mentalizing ability due to delayed development based on chronological age. Additionally, the participants in our research presented a wider age range compared to Sivaratnam et al. (2012). On the basis of these results, the SIPI represents a useful instrument to support the ASD diagnosis. Specifically, the SIPI assesses the ability to correctly interpret the presented social scenarios (interpretation), “put oneself in another’s shoes” (response generation), and determine whether other people’s social behaviors are right or wrong (response evaluation).

Our results regarding the SIPI are consistent with a previous study that demonstrated differences between ASD and TD children on this task (Pino et al., 2018). Additionally, Mazza et al. (2017) showed that mentalizing ability plays a key role in the development of social abilities, and the lack of ToM competences in children with ASD impairs their competent social behavior (Mazza et al., 2017). Thus, these components are closely related and improved mentalizing ability might also enhance social behavior.

Collection of the data examined in this study should allow clinicians to plan a treatment focused on social abilities to improve the relationship with other people and avoid isolation and the emergence of other clinical symptomatology, such as depression or anxiety disorder. Furthermore, the systematic use of SC measures in clinical evaluations might help monitor improvements related to treatment and therapy.

In conclusion, we think that the data provided in this study are valuable because they emphasize the utility of incorporating SC measures into diagnostic processes in ASD clinical practice. In particular, the SIPI showed valid accuracy in distinguishing between ASD and TD children. These findings indicate that this test can be implemented into the diagnostic procedure. Additionally, the data provided by our work suggest the cutoff points for each of the examined SC tests (Table 3); these data should allow examiners to use these tests with normative values.

We are aware that the present study has some limitations. (1) Our two samples differed in chronological age. However, we stress that the development of SC competencies, particularly mentalizing ability, is related more to mental rather than to chronological age (Pino et al., 2018). (2) This study is also limited by the small Italian sample size; future studies are needed to demonstrate the generalizability of our results. (3) Performance would also need to be compared to other clinical conditions to determine whether these tasks adequately discriminate autism from competing diagnoses. Given that other clinical conditions also present with impairments in social performance, it is necessary to investigate the utility of these tasks for selectively aiding an ASD diagnosis.

## Data Availability Statement

The datasets generated during the current study are not publicly available because the data were obtained in the course of mental health care. However, they are available from the corresponding author on reasonable request.

## Ethics Statement

The studies involving human participants were reviewed and approved by the Ethics Committee approved the protocol (number 186061/17) prior to the recruitment of participants, according to the principles established by the Declaration of Helsinki. Written informed consent to participate in this study was provided by the participants’ legal guardian/next of kin.

## Author Contributions

MM and MP designed the research. RV, MA, and CD collected the data. FM, MP, and MV analyzed the data. All authors contributed to writing the manuscript.

## Conflict of Interest

The authors declare that the research was conducted in the absence of any commercial or financial relationships that could be construed as a potential conflict of interest.

## Abbreviations

ADOS-2: Autism Diagnostic Observation Schedule–Second Edition
ASD: autism spectrum disorder
AUC: area under the curve
CST: Comic Strip Task
DSM-5: *Diagnostic and Statistical Manual of Mental Disorders, Fifth Edition*
ROC: receiver operating characteristic
SC: social cognition
SIPI: Social Information Processing Interview
TD: typically developing
ToM: Theory of Mind
TROG-2: Test for Reception of Grammar–Second Edition
VMA: verbal mental age
