# Supplementary material for: Validity of Social Cognition Measures in the Clinical Services for Autism Spectrum Disorder
Source: Front Psychol. 2020 Feb 5;11:4. doi: 10.3389/fpsyg.2020.00004 (PMC7012901; doi:10.3389/fpsyg.2020.00004)
Supplement: Supplementary file 1 [file Data_Sheet_1.PDF]

APPENDIX

ITALIAN EXAMPLE OF A SOCIAL INFORMATION PROCESSING INTERVIEW-SIPI STORY

ESEMPIO: STORIA E "*IL GIOCO DELLE COSTRUZIONI*".

<u>Descrizione</u>

Social Information Processing Interview (SIPI; Ziv & Sorogon, 2011; Ziv et al., 2014) è un'intervista semi-strutturata che mira a valutare la percezione dei bambini in situazioni sociali rappresentate con i loro pari. In particolare, l'intervista è stata ideata per identificare gli errori di attribuzione nella percezione delle intenzioni sociali altrui. Sotto forma di libro di racconti, al bambino vengono presentate una serie di vignette che raccontano la storia di un orsetto (Michael nella versione per bambini e Lisa nella versione per bambine), che interagisce con due coetanei o con la madre. L'intervista è strutturata nel seguente modo:

- Identificazione delle emozioni a partire dalle espressioni facciali;
- Due storie in cui il comportamento dell'orsetto viene interrotto dall'azione di un compagno;
- Due storie in cui l'orsetto prova ad unirsi a due compagni che stanno giocando;
- Una storia in cui l'orsetto interagisce con la madre.

<u>Punteggi e valori normativi</u>

Al fine di rendere più agevole la comprensione del test e l'assegnazione dei punteggi, di seguito viene riportata la Storia E: "*Il gioco delle costruzioni*".

"Ora ti racconterò un po' di storie che riguardano un orsetto di nome Michael (Figura 1). Lui indossa una maglietta scura ed è in tutte le storie che io ti racconterò. Ti farò alcune domande sulle storie. Non ci sono risposte giuste o sbagliate. Voglio solo sapere cosa pensi. Sei pronto?"

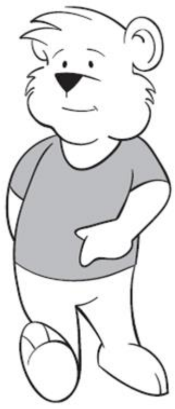

Figura 1. *Orsetto Michael, protagonista delle storie del SIPI*

"In questa storia, due orsetti stanno giocando con le costruzioni. L'orsetto 1 dice: <<Queste costruzioni sono divertenti!>>. L'orsetto 2 risponde: <<Si.. Sai, anche Michael vuole giocare con le costruzioni>>.

Michael sta guardando gli altri bambini che giocano" (Figura 2).

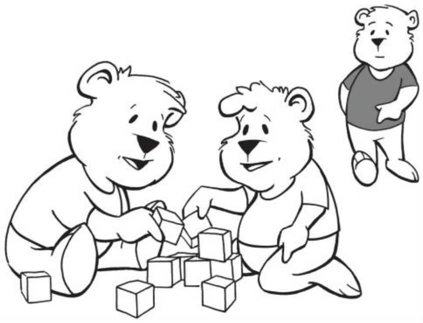

Figura 2

"Michael si avvicina ad uno degli orsetti e chiede: <<Posso giocare con te?>>. L'orsetto 2 risponde:<<Mi dispiace, l'insegnante ha detto che solo due persone alla volta possono giocare con le costruzioni>>" (Figura 3).

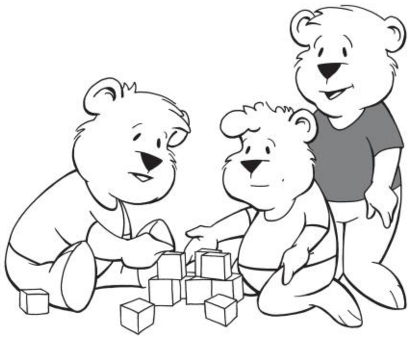

Figura 3

1. Codifica delle informazioni

La sotto-componente "codifica" valuta il livello di dettagli che il bambino ricorda riguardo le 4 storie. Al bambino viene chiesto: *"Ora dimmi cos' è successo dall'inizio di questa storia fino alla fine".*

Il punteggio viene assegnato nel modo seguente:

- Completo (1 punto) se il bambino descrive tutte le parti della storia originale;
- Incompleto/con aggiunte (0 punti) se una parte della storia è mancante o parti della storia sono sbagliate o il bambino aggiunge eventi che non fanno parte della storia originale.

Se il bambino mostra delle difficoltà nel raccontare spontaneamente, viene aiutato attraverso una serie di domande relative a ciascuna parte della storia. Ad esempio, per la parte iniziale si chiede:

- *I bambini stanno giocando con le costruzioni?*

Se il bambino risponde di NO procedere con altre due domande:

- *I bambini stanno giocando fuori con la palla?*
- *I bambini stanno colorando delle immagini?*

2. Interpretazione delle informazioni

Questa sotto-componente valuta l'attribuzione ostile al comportamento degli altri.

Al bambino viene chiesto:

- *"Pensi che i bambini che non lasciano giocare Michael sono cattivi oppure no?"*

Viene attribuito punteggio 0 se il comportamento dei bambini viene interpretato come non ostile; al contrario, viene assegnato punteggio 1 se il comportamento viene interpretato come ostile.

3. Costruzione della risposta

Il punteggio relativo a questo step deriva dalle risposte del bambino alla seguente domanda:

- *"Cosa faresti o diresti al posto di Michael se tu volessi giocare con i tuoi compagni e loro ti dicessero che solo due alla volta possono usare le costruzioni?"*

L'esaminatore codifica la risposta del bambino come "competente" o "non competente", (punteggi più elevati rappresentano livelli più alti di ostilità attribuita, in un range che va da 0-8; per maggiori dettagli relativi alle codifiche consultare Ziv e Sorogon, 2011; Mazza et al., 2017; Pino et al., 2018).

4. Valutazione della risposta

Gli item di “valutazione della risposta” esaminano il modo in cui il bambino valuta il comportamento di altre persone, in termini di giusto e sbagliato; i punteggi sono ottenuti da una combinazione di 36 risposte totali (4 storie x 3 componenti x 3 domande).

Al bambino vengono mostrate tre differenti reazioni di Michael alla situazione: competente, ostile ed evitante.

- PRIMO SCENARIO (Competente)

“Michael potrebbe dire: <<Allora posso giocare al prossimo turno?>>”

A questo punto chiedere al bambino:

- *“Quello che chiede Michael è giusto o sbagliato?”*
- *“Pensi che agli altri bambini farebbe piacere quello che chiede Michael?”*
- *“Pensi che gli altri bambini lascerebbero giocare Michael se lui dicesse questo?”*

- SECONDO SCENARIO (Aggressivo)

“Michael potrebbe buttare giù tutte le costruzioni e dire agli altri bambini: <<Se io non posso giocare allora non potete giocare neanche voi!>>.

A questo punto chiedere al bambino:

- *“Quello che fa Michael è giusto o sbagliato?”*
- *“Pensi che agli altri bambini farebbe piacere quello che fa o dice Michael?”*

- *"Pensi che gli altri bambini lascerebbero giocare Michael se lui si comportasse in questo modo?"*

- TERZO SCENARIO (Evitante)

"Michael potrebbe piangere e dire: <<Non è giusto!>>

Chiedere al bambino:

- *"Quello che fa Michael è giusto o sbagliato?"*
- *"Pensi che agli altri bambini farebbe piacere quello che fa o dice Michael?"*
- *"Pensi che gli altri bambini lascerebbero giocare Michael se lui si comportasse in questo modo?"*
